# How Vacations Affect Parkinson's Disease

**DOI:** 10.1002/mdc3.13597

**Published:** 2022-11-02

**Authors:** Jules M. Janssen Daalen, Jessica Hubbers, Mirmohsen Sharifi Bonab, Soania Mathur, Dick H.J. Thijssen, Bastiaan R. Bloem, Marjan J. Meinders

**Affiliations:** ^1^ Center of Expertise for Parkinson and Movement Disorders, Department of Neurology Radboud University Medical Center, Donders Institute for Brain, Cognition and Behavior Nijmegen The Netherlands; ^2^ Department of Research and Innovation Helse Fonna Haugesund Norway; ^3^ Clinical Research Development Unit of Tabriz Valiasr Hospital Tabriz University of Medical Sciences Tabriz Iran; ^4^ UnshakeableMD Oshawa Ontario Canada; ^5^ Department of Physiology Radboud University Medical Center Nijmegen The Netherlands; ^6^ IQ Healthcare, Radboud Institute for Health Sciences Radboud University Medical Center Nijmegen The Netherlands

**Keywords:** Parkinson's disease, symptoms, determinants, symptom severity, vacation, travel

Parkinson's disease (PD) is a neurodegenerative disorder that is characterized clinically by a variety of motor and nonmotor symptoms with debilitating effects on quality of life. Although the disease is gradually progressive, symptoms vary from day to day, which significantly influences daily functioning.^1^ Variations across consecutive days are likely attributable to contextual factors of daily life, such as changes in physical activity, sleep, stress, or diet.^2^, ^3^ Anecdotal evidence is beginning to suggest that environmental factors, such as high‐altitude stays or warmer temperatures, may also affect symptom severity in people with PD (PwP). However, the influence of such environmental factors has thus far not been studied systematically. A better understanding of how contextual factors drive variations in symptom severity might offer new therapeutic perspectives and could facilitate personalized counseling.

To quantify and clarify the influence of contextual factors on PD symptom severity, we performed an international survey (Design in Supplementary Materials 1) to capture the symptomatic experiences of PwP during a vacation. The vacation context provides a unique “natural experiment” and an excellent opportunity for studying the role of contextual factors on intraindividual changes in symptom severity, as much of an individual's natural context changes while on vacation. In addition, the vacation period provides an opportunity to compare the effect sizes of various different contextual factors, which all change concurrently during a vacation. Lastly, although vacation and travel are important contributors to quality of life, vacation experiences have only sparsely been investigated.

The primary purpose of our study was to explore what contextual factors were associated with short‐term symptom changes. Global symptom changes as well as a selection of cardinal symptoms were rated on a 3‐point ordinal scale (improvement, unchanged, or worsening). A secondary purpose was to explore the patterns of changes in symptom severity in this different context using principal component analysis.

In total, 212 participants completed the survey, of whom 65 did not go on a vacation in the past 3 months. Analyses were conducted on the recent vacation subgroup of 147 individuals (population characteristics: Table S1, Supplementary Materials 2). While on vacation, participants most frequently reported increased physical activity (61.1%) and a decrease in stress (50.7%). Apart from 44.6% reporting an increased alcohol intake, diet and medication intake usually remained unchanged (Table S2, Supplementary Materials 3).

Almost half of the population (44.9%) reported global symptom improvement on vacation, whereas 12.9% noted worsening of symptoms. Improvements in motor symptoms mainly related to tremor, stiffness, and pain/cramps, whereas nonmotor symptoms that improved included mood, limitations in daily life, and fatigue (Figure S1, Supplementary Materials 3). Individuals who reported global symptom improvement were younger (median age, 60 years; range, 40–78 years) than those who reported no change or worsening of global symptoms (median age, 66 years; range, 44–77 years; *P* = 0.0012), but there were no between‐group differences in disease duration (*P* = 0.96), Parkinson's Disease Questionnaire–8 score (*P* = 0.34), or sex (*P* = 0.80). Individuals who went on vacation for more than a week did not report global symptomatic improvement more often than individuals with shorter stays (*P* = 0.21).

More physical activity and more sleep were significantly associated with improved global symptoms, whereas stress reduction was not (Table 1). Reductions in physical activity and more stress were significantly associated with worsening of global symptoms, but reduced sleep was not (Table S3, Supplementary Materials 3). Specifically, physical activity was associated with improvements in walking, pain/cramps, and slowness. More sleep was associated with a wide range of motor and nonmotor symptoms. High‐altitude stay was associated with less stiffness, whereas stress reduction was not significantly associated with improvement in specific symptoms (Table 1). Of the individuals with symptom improvement on vacation, 64% reported a beneficial influence of warmer temperatures. In this subgroup, pain/cramps, stiffness, and limitations in daily life were most often improved (not shown). Reduced physical activity was associated with a concurrent worsening of the very same symptoms that improved among those whose physical activity increased, suggesting a dose–response relation (Table S3, Supplementary Materials 3). More stress was primarily associated with worsening of tremor, fatigue, sleep, and slowness, and less sleep was associated with worsening fatigue and mood. Individuals who reported global symptom worsening attributed to (too) warm temperatures (n = 11) reported worsening of pain/cramps (36%), walking (45%), and stiffness (45%).

**TABLE 1:** Effect estimates for potential determinants of symptomatic improvement

Determinants	High Altitude (n = 21)				More Physical Activity (n = 88)				More Sleep (n = 49)				Less Stress (n = 71)			
	Δ %	OR	95% CI		Δ %	OR	95% CI		Δ %	OR	95% CI		Δ %	OR	95% CI	
Primary outcome																
Global symptoms	21.2	2.1	0.8	5.8	25.4	**3.4**	**1.4**	**8.5**	27.9	**3.1**	**1.4**	**7.2**	6.5	1.5	0.7	3.2
Secondary outcomes																
Tremor	11.0	1.5	0.5	4.7	15.8	2.0	0.7	5.8	18.1	1.7	0.6	4.5	−1.4	0.9	0.4	2.4
Walking	15.7	2.0	0.7	6.4	28.9	**5.0**	**1.4**	**17.4**	34.5	**5.7**	**1.9**	**16.9**	4.5	1.4	0.6	3.4
Balance	10.6	1.5	0.5	4.8	11.6	1.8	0.6	5.4	27.7	**3.4**	**1.2**	**9.7**	−0.2	1.1	0.4	2.7
FMS	19.1	2.4	0.7	8.4	12.9	3.1	0.9	11.4	20.5	**2.9**	**1.0**	**8.7**	−7.9	0.8	0.3	2.0
Pain	8.0	1.5	0.5	4.2	25.3	**4.0**	**1.4**	**11.5**	31.4	**4.1**	**1.6**	**10.5**	7.4	1.6	0.7	3.6
Dyskinesia	−10.5	0.6	0.1	3.3	25.7	3.8	0.9	17.0	22.3	2.4	0.6	9.2	−10.1	0.6	0.2	1.8
Stiffness	26.8	**2.9**	**1.0**	**8.2**	7.5	1.5	0.6	3.8	21.3	2.0	0.8	4.8	4.8	1.4	0.6	3.2
Slowness	16.8	1.9	0.7	5.7	22.9	**3.7**	**1.3**	**11.0**	21.6	**2.4**	**1.0**	**5.9**	−15.0	0.6	0.2	1.3
LDL	−0.2	0.9	0.3	2.8	26.4	**3.5**	**1.1**	**10.9**	26.9	**3.3**	**1.2**	**9.0**	5.9	1.3	0.5	3.2
Sleep	4.6	1.0	0.3	3.3	−9.9	0.9	0.3	2.3	53.9	**11.9**	**3.9**	**36.0**	4.0	1.3	0.6	3.1
Mood	−2.6	0.9	0.3	3.0	16.8	2.6	0.8	8.7	38.8	**5.1**	**1.7**	**15.4**	−3.7	0.9	0.4	2.3
Fatigue	−7.5	0.8	0.2	2.4	9.7	1.5	0.6	3.8	8.5	1.3	0.6	3.2	12.2	2.0	0.9	4.6

For every association, Δ % and OR are given. Δ % reflects the percentage point difference of symptom improvement between the group that was exposed to the potential determinant and the group with unchanged determinant exposure (compared with at home). Statistically significant findings are underlined and shown in bold. Associations for other determinants can be found in Table S3 (Supplementary Materials 3).

Abbreviations: OR, odds ratio; CI, confidence interval; FMS, fine motor skills; LDL, limitations in daily life.

Principal component analysis (Table S4, Supplementary Materials 4) identified the following 4 clusters of symptom improvement: medication (im)balance, dopaminergic state, physical and mental load, and (over)activity. Clusters and all reported symptom changes and associations are visualized in Figure S2 (Supplementary Materials 4).

In this article, we identified multiple associations between contextual factors of daily life and motor and nonmotor symptoms, yielding important insights in the drivers of day‐to‐day varying symptom severity. Findings on physical activity and sleep are in accordance with previous studies that investigated their symptom‐specific effects.^2^ Interestingly, reduced stress while on vacation was not associated with symptomatic improvement, although increased stress resulted in global symptom worsening. It is well known that stress predominantly increases tremor, but our results align with a recent survey that demonstrated a widespread symptomatic impact of stress.^3^ The fact that tremor was least associated with other symptoms might reflect the complexity of tremor pathophysiology, as indicated by the multiple networks and neurotransmitters involved.^4^ Global symptom improvement associated with warmer temperatures is often reported by PwP, but we are not aware of previous research reporting this effect. It is unknown whether this is caused by a direct physical effect (eg, less stiffness, cramps) or by indirect effects. For example, autonomic dysfunction causes dysfunctional thermoregulation, which is more noticeable in colder climates.^5^ The anecdotal evidence of symptomatic improvement at high altitudes in PwP, corroborated by this study, requires further elucidation.

An important limitation of this study is the risk of reversed causality occurring when PwP adjust their vacations to the beneficial effects on symptom severity. Selection bias might have occurred, as going on vacation is not possible for some PwP, such as those with more advanced disease. Nonetheless, this “natural experiment” provides a universal recipe for a PD‐friendly lifestyle, consisting of engaging in regular physical activity and stress‐reducing activities; avoiding physical and mental overload; and adhering to a regular, uncompromised sleep schedule and structured daily routine. As such, the findings of the present study may help PwP to minimize avoidable symptom burden both on vacation and at home.

## Author Roles

(1) Research Project: A. Design, B. Execution, C. Data Processing, D. Analysis; (2) Manuscript: A. Writing, B. Editing of Final Version of the Manuscript.

J.M.J.D.: 1C, 1D, 2A, 2B

J.H.: 1A, 1B, 1C, 2B

M.S.B.: 1A, 1B, 2B

S.M.: 1A, 1B, 2B

D.H.J.T.: 1D, 2A, 2B

B.R.B.: 1A, 2B

M.J.M.: 1A, 1B, 1C, 1D, 2A, 2B

## Disclosures

**Ethical Compliance Statement:** The research ethics committee approved the study protocol (Medische Ethische Toetsingscommissie (METC) East Netherlands, file number 2018‐4405). Participants in the survey gave (digital) informed consent prior to completing the survey by checking a tick box agreeing to the terms and conditions of participation. Survey results were collected in an anonymized manner. We confirm that we have read the Journal's position on issues involved in ethical publication and affirm that this work is consistent with those guidelines.

**Funding Sources and Conflicts of Interest:** This study was supported by The Michael J. Fox Foundation for Parkinson's research within the Therapeutic Pipeline Programme (Grants 14548 and 019201). The Centre of Expertise for Parkinson and Movement Disorders was supported by a Center of Excellence grant by the Parkinson's Foundation. The authors report no conflict of interest.

**Financial Disclosures for the Previous 12 Months**: Professor Bloem serves as the co‐Editor in Chief for the *Journal of Parkinson's Disease*; serves on the editorial board of *Practical Neurology and Digital Biomarkers*; has received fees from serving on the scientific advisory board for UCB, Kyowa Kirin, Zambon and the Critical Path Institute (paid to the institute); has received fees for speaking at conferences from AbbVie, Biogen, UCB, Zambon, Roche, GE Healthcare, Oruen, and Bial (paid to the institute); and has received research support from the Netherlands Organization for Health Research and Development, The Michael J. Fox Foundation, UCB, the Stichting Parkinson Fonds, Hersenstichting Nederland, de Stichting Woelse Waard, Stichting Alkemade‐Keuls, de Maag Lever Darm Stichting, Parkinson NL, Davis Phinney Foundation, the Parkinson's Foundation, Verily Life Sciences, Horizon 2020, the Topsector Life Sciences and Health, Nothing Impossible, and the Parkinson Vereniging outside the submitted work. Jules M. Janssen Daalen, Jessica Hubbers, Mirmohsen Sharifi Bonab, Soania Mathur, Dick H.J. Thijssen, and Marjan J. Meinders have nothing to disclose.

## Supporting information


**Supplementary Materials 1**. Study Design and Vacation Survey

**Supplementary Materials 2**. Demographics and Vacation Characteristics

**Supplementary Materials 3**. Symptom Changes, Changes in Contextual Factors, and Determinants of Symptomatic Worsening

**Supplementary Materials 4**. Principal Component Analysis and Visualization of Study Results
