# Supplementary material for: How Vacations Affect Parkinson's Disease
Source: Mov Disord Clin Pract. 2022 Nov 2;10(1):151–3. doi: 10.1002/mdc3.13597 (PMC9847288; doi:10.1002/mdc3.13597)
Supplement: Supplementary file 1 — Supplementary Materials 1. Study Design and Vacation Survey [file MDC3-10-151-s001.docx]

# Supplementary Materials 1: Design and Survey

Together with individuals with PD, a survey was designed in a consensus-based manner. An important source of inspiration for the survey was an earlier patient-researcher-led survey that investigated what symptoms affect quality of life, including the role of contextual factors, in relation to the willingness to self-monitor those symptoms.^1^ The selection of symptoms was influenced by clinical experience and earlier vacation-related symptom experience of one patient advisor.

The survey consisted of four elements (Supplementary Materials 1). First, baseline demographics (age, gender, place of residency) and disease characteristics (disease duration and disease-affected quality of life measured with the Parkinson’s Disease Questionnaire-8, PDQ-8^2^ were collected. The survey also captured several environmental aspects associated with the vacation (altitude and climate at the vacation destination) and any change in lifestyle during vacation compared to during normal daily living at home, including physical activity, sleep, stress, diet and changes in medication intake. All items were rated on a three-point ordinal scale (more, equal, less). More detailed vacation characteristics, such as number of days in certain locations and average temperatures, returned many missing values. Therefore, these data were excluded from the analysis altogether.
Questions concerning diet specifically addressed the intake of carbohydrates, protein and alcohol. Stress levels during the vacation were assessed using the Perceived Stress Scale (PSS).^3^ Subsequently, participants gave an impression of the subjective change in two separate categories: firstly, the change in global symptoms (primary outcome), and secondly, the change in specific motor and non-motor PD symptoms while on vacation compared to at home (secondary outcome). These changes were again scored on a three-point ordinal scale (improved, unchanged, worsened).

Individuals that indicated symptom improvement or worsening while on vacation were additionally asked whether the environmental temperature (warm or cold) might have contributed to this change. As this was only asked to individuals with global symptom improvement on vacation, temperature was analyzed separately from the other potential determinants. Sleep was a contextual factor as well as an outcome, but sleep as a contextual factor evaluated sleep duration, whereas sleep as outcome evaluated sleep quality (worsened, unchanged, improved). Finally, participants were invited to describe (in their own words) the self-perceived change and cause of change in PD symptoms in a free-text field.

## Recruitment

Participants were recruited from October 2018 until September 2019, as to include responses during multiple seasons to account seasonal variation in symptoms. However, nearly all responses occurred during the summer, and therefore, we could not account for seasonal variation in PD symptoms in our analyses. The survey was distributed through multiple channels, for which we collaborated with patient organizations and representatives worldwide. The survey was distributed via various platforms: ParkinsonNEXT, a platform for patient participation in research in the Netherlands, via the European Parkinson’s Disease Association (EPDA), the Norwegian patient organization, and the Parkinson’s Foundation, who distributed the survey via their Centers of Excellence, and via social media channels of the authors. An online survey tool, SurveyGizmo, was used to collect the data from participants. Lastly, at Tabriz University of Medical Sciences in Iran, a patient information event was organized by one of the authors (MSB) and a paper-version was handed out. To avert language barriers, the survey was available online in Dutch, English, Norwegian and in Farsi as a paper-based survey.

## Participants

Participants were at least 18 years of age, and had a self-reported PD diagnosis. For the purpose of this study and for bias reduction (see ‘Bias’), we included only those participants who went on vacation recently, meaning in the 3 months before survey completion. A vacation was defined as being away from home, with at least one overnight stay.

## Bias

Confirmation bias was limited by avoiding direct suggestive effects of potential determinants on symptoms and by inquiring about a large variety of contextual factors. We asked for experiences during the most recent vacation (maximum 3 months) because of the risk of recall bias, as more recent experiences can be assumed to have better reliability. Recall bias was additionally mitigated by encouraging the respondent to complete the survey together with their partner who joined the respondent during the vacation, if applicable. Asking participants about changes that occurred during any type of vacation would keep individuals blinded for the effects of the different vacation-related factors, such as a change in altitude or change in environmental temperature.

## Outcome measures and data analyses

The primary outcome measure was the global symptomatic change (improvement, unchanged or worsening) while on vacation as reported by subjects after return from their vacation. The secondary outcome was change of specific symptoms. Both outcomes were scored on the aforementioned 3-point ordinal scale (improved, unchanged, worsened).

These outcomes were compared to determinants, also scored on a 3-point ordinal scale (more, equal, less). Because the distance between the three points on the scale is unknown and there is not necessarily a linear dose-response relation between determinant and symptom, we avoided direct comparisons between the two subgroups in which exposure to the determinant changed (‘more’ and ‘less’). Therefore, we always used the ‘equal’ subgroup as a reference for symptom changes and compared that to symptom changes in either the subgroup with 'more‘ or ‘less’ exposure to one specific determinant. As all individuals went on vacation, the effect of vacation itself is not part of the equation.
The between-group percent point difference (Δ%) was calculated for both the between-group difference in symptom improvement and symptom worsening. The Δ% is calculated between the group with beneficial change in determinant exposure (for example, more physical activity) and the reference (equal exposure to the determinant). The same was done for symptom worsening, but then for the Δ% between a disadvantageous change in determinant exposure (e.g. less sleep) and reference. For the same association analyses, logistic regression analyses were conducted. All regression analyses were corrected for age, gender, disease duration and PDQ-8. Odds ratios (OR) for odds of symptomatic change (any significant improvement or worsening relative to unchanged symptoms) and 95% confidence intervals were displayed as summary statistics. As tertiary analysis, clustering of symptoms (those that tended to improve together intra-individually) were analyzed using principal component analysis (PCA). Varimax rotation using Kaiser normalization was applied on the component matrix. Eigenvalues greater than one were considered significant. To summarize all findings of associations between contextual factors and symptom improvement, we designed a figure that illustrates the symptom clusters, the average difference of specific symptoms on vacation as well as the association strength (OR) between contextual factors and symptoms.

Descriptive statistics were used for baseline variables, contextual factors and confounders. Between-group differences in baseline variables between the subgroup that reported global symptom improvement and those with unchanged or worsening of symptoms was conducted using Mann-Whitney tests not assuming equal variances (suitable as demonstrated by Levene’s test for equality of variance). Missing data were prevalent, often due to not recalling, but data were not imputed. If participants did not experience a particular symptom, or if they could not remember the influence of a factor on a specific symptom, they were excluded from that sub-analysis. All quantitative data analyses were performed in SPSS v24. Because of the explorative nature of this study, no correction of the statistical significance threshold was applied and a p-value of 0.05 was considered significant.

## References

1. Mathur S, Mursaleen L, Stamford J, DeWitte S, Robledo I, Isaacs T. Challenges of Improving Patient-Centred Care in Parkinson's Disease. J Parkinsons Dis 2017;7(1):163-174.
2. Jenkinson C, Fitzpatrick R, Peto V, Greenhall RCD, Hyman N. The PDQ-8: Development and validation of a short-form parkinson's disease questionnaire. Psychology & Health 1997;12:805-814.
3. Cohen S, Kamarck T, Mermelstein R. A global measure of perceived stress. J Health Soc Behav 1983;24(4):385-396.

## Vacation Survey

Thank you for your interest in this survey about vacation! Vacation can have a huge effect on Parkinson’s disease symptoms. There are fascinating stories of remarkable changes in the symptoms of Parkinson’s when people go on vacation. However, this has never been studied systematically. For us it is important to know more about vacation experiences, as this can provide new insights into (new treatments for) Parkinson’s disease.

We want to learn from your experiences! If you were going on vacation with your spouse, a friend or anyone else close to you, please try to complete this survey together. If that does not work, or you were on vacation without acquaintances, you can complete the survey on your own.

**Part I: About yourself**

First, we ask some general questions.

1. What is your age? [open] years
2. What is your gender?

- Male
- Female

1. In which city do you live? [open]
2. In which state do you live?
3. In which year were you diagnosed with Parkinson’s disease? [open]*.*

The following questions are a validated way to ask you about your Parkinson’s disease symptoms.

1. [PDQ-8] Due to having Parkinson’s disease, how often during the last month have you…

*Please* ***tick one box*** *for each question*

[Never/Occasionally/Sometimes/Often/Always, or cannot do at all]

1. Had difficulty getting around in public?
2. Had difficulty dressing yourself?
3. Felt depressed?
4. Had problems with close personal relationships?
5. Had problems with your concentration, e.g. when reading or watching TV?
6. Felt unable to communicate with people properly?
7. Had painful muscle cramps or spasms?
8. Felt embarrassed in public due to having Parkinson’s disease?
9. **In general**, what is the effect of physical activity on your Parkinson’s symptoms? By this we mean, for example, the effect you experience when you are active, directly after being active or in between two activities.

- Symptoms increase.
- Symptoms decrease.
- Physical activity does not have an effect on my Parkinson’s symptoms.

1. **In general**, what does a warm ambient temperature do to your Parkinson’s symptoms?

- Symptoms increase
- Symptoms decrease
- A warm ambient temperature does not have an effect on my Parkinson's symptoms

1. **In general**, what does a cool ambient temperature do to your Parkinson’s symptoms?

- Symptoms increase
- Symptoms decrease
- A cool ambient temperature does not have an effect on my Parkinson's symptoms

1. **In general**, what is the effect of stress on your Parkinson’s symptoms? By this we mean stress due to, for example, situations at home or at work.

- Symptoms increase
- Symptoms decrease
- Stress does have an effect on my Parkinson’s symptoms.

1. Have you **ever** experienced a difference in your Parkinson’s symptoms while staying at high altitude, for example, in the mountains?

- Yes, at least once I experienced a very clear decrease in my symptoms while staying at high altitude
- Yes, at least once I experienced a very clear increase in my symptoms while staying at high altitude
- No, I have never experienced a clear change in my symptoms while staying at high altitude
- I do not know, because I have never been at high altitude
- I do not know, because I have forgotten how I felt at high altitude

1. [if previous question was yes(1,2)]: You indicated that you have experienced a difference in Parkinson’s symptoms while staying at high altitude. Can you please describe in which difference(s) you experienced, in your own words? Be as detailed as possible.

**Which vacation experiences would you like to share with us?**

This survey is now divided into two parts. The first part is about your recent vacation experiences between July and September 2018.

Please select the option that is most applicable to your situation and wishes.

1. When did you go on vacation? This may have been a longer break, or only a weekend, during which you did not sleep at home for at least two consecutive nights.

- I have been on vacation between July and September 2018
- I have been on vacation between June 2016 and June 2018
- I have been on vacation in both periods (June 2016 – June 2018 as well as July – September 2018) and I would like to share my experiences from the recent vacation
- I have not been on vacation in one of these periods 🡪 You indicated that you neither have been on vacation between June 2016 and June 2018 nor between July and September 2018. Unfortunately you are not eligible to fill out the rest of the survey.

**About your recent vacation experiences**

You indicated that you have been on vacation between July and September 2018. Please try to remember the vacation in this period. It may have been a longer vacation, of a weekend trip, during which you did not sleep at home for at least two consecutive nights. The following questions are about this vacation.

1. What was your vacation destination? Please name the city, state (if applicable) and country. If you have visited multiple vacation destinations (for example, if you visited multiple cities in one trip), you can fill out multiple destinations.
   When you stayed at the same vacation destination the entire vacation, just name the place, (province/state if applicable,) and country for “destination 1” only.
2. During what period did you go on vacation? DD-MM-2018 / DD-MM-2018
3. What was the average temperature during this vacation? [number] °C/F.
4. In what kind of environment did you stay during this vacation? Multiple answers are possible (for example, if you have visited multiple places).
   1. Mountains
   2. Beach
   3. City
   4. Forest
   5. Countryside
   6. Other, namely: …………………
5. [If respondent stayed in multiple environments]:
6. [if you stayed in the mountains]: How long did you stay in the mountains? X days [number]
7. [if you stayed on the beach]: How long did you stay on the beach? X days [number]
8. [if you stayed in a city]: How long did you stay in the city? X days [number]
9. [if you stayed in the forest]: How long did you stay in the forest? X days [number]
10. [if you stayed at the countryside]: How long did you stay at the countryside? X days [number]
11. [if you stayed in another environment]: How long did you stay here? X days [number]

**Page: your Parkinson’s symptoms**

1. Did you, or someone else who was with you during this vacation, notice if your Parkinson’s symptoms changed during the vacation, compared to how you normally experience your symptoms at home? Please do not only think of differences in motor symptoms, such as tremor, slowness, balance, difficulty walking or fine motor skills (such as getting dressed or writing), but also report on non-motor symptoms such as pain, mood or sleep and your overall functioning.
   1. Yes, I clearly experienced more Parkinson’s symptoms during my vacation.
   2. Yes, I clearly experienced less Parkinson’s symptoms during my vacation.
   3. No, I did not experience any or only very small differences in my Parkinson’s symptoms during my vacation.
2. We will now ask a couple of questions about changes in specific Parkinson’s symptoms, which you or someone close to you may have noticed changed during your vacation. Below you will find a list of symptoms. Indicate for every symptoms to what extent the symptom was different during the vacation, compared to how you normally experience that symptom at home. If you do not recall a particular symptom or a symptom is not applicable because you normally don’t experience it, please indicate so in your answers.

Did you experience changes in: [Yes, I experienced this symptom less/Yes I experienced this symptom more/No, this symptom did not change/I do not recall/Not applicable because I do not experience this symptom normally]

- 1. Tremor
  2. Gait problems
  3. Balance problems
  4. Difficulties with fine motor skills (i.e., writing, getting dressed)
  5. Pain or muscle cramps
  6. Uncontrolled movements (no tremor)
  7. Stiffness of the muscles
  8. Overall slowness

1. We are also interested in changes of health problems, which you or someone close to you may have noticed during your vacation. Please indicate for each health problem listed below to what extent it was different during the vacation, compared to how you normally experience that problem at home.[yes I experience this health problem less/yes, I experience this health problem more/ No this health problem was not different / I do not recall/ Not applicable because I do not have this health problem normally]
   1. General restrictions of daily activities
   2. Difficulty sleeping
   3. Changes in mood
   4. Fatigue
2. [if at least one item is scored as more/less/better/worse in the previous 2 questions]: You indicated to have experienced a change in at least one symptom or health problem during the vacation. Can you please describe which change(s) you experienced in your own words? Be as detailed as possible.
3. What do you think caused this change in your Parkinson’s symptoms? [Open]
4. To what extent was the environment you stayed in, for example, the beach, mountains or a city, of influence on the changes you experienced?

- a very great extent
- a great extent
- some extent
- Not at all
- I do not recall

1. You indicated that the environment you were in was of influence on the changes in Parkinson’s symptoms. Can you please describe, in your own words, why you think so.[open]
2. How long did this/these change(s) typically last?
   1. 0-30 minutes
   2. 30-60 minutes
   3. Several hours
   4. A day
   5. Several days or longer
   6. Variable duration
3. Have you experienced these changes just once, or multiple times while you were on vacation?
   1. Once
   2. It happened more often
   3. I do not recall

[If destination = Mountains] You indicated earlier that you visited the mountains during your vacation. The next questions are about the time that you spent in the mountains:

1. [if destination= mountains] To what extent did your Parkinson’s symptoms change when you stayed in the mountains, compared to how you experience your symptoms at home?
   1. Symptoms increased
   2. Symptoms decreased
   3. I did not experience a clear difference
   4. I do not recall
2. [If previous question = yes] You indicated that you experienced a difference in symptoms while staying in the mountains. Can you please describe, in your own words, what your experiences were? Be as detailed as possible. [open]
3. During your vacation, have you been at an altitude above 6500 feet? It does not matter if this was a short or long stay. Passing through a high mountain pass by car already is sufficient. A longer stay at high altitude is also possible.
   1. Yes, I was at an altitude of 6500 feet or higher
   2. Yes, I was at an altitude of 6500 feet or higher, but only on an airplane
   3. No, I have not been at an altitude above 6500 feet
4. [if Q30 = Yes(option 1)] You indicated that you were at 6500 feet or above. Did you experience a change in Parkinson’s symptoms while staying at this high altitude?

- Yes, symptoms increased
- Yes, symptoms decreased
- No, I did not experience a change in symptoms
- I do not recall

1. [if destination = beach] Previously you indicated that you went to the beach. Did you go scuba diving during your stay at the beach?

- yes, I went scuba diving
- No, I did not go scuba diving

1. [If 32=Yes] Did you experience a change in Parkinson’s symptoms when you went scuba diving?

- Yes, my symptoms increased
- Yes, my symptoms decreased
- No, I did not experience a change in symptoms

1. [If 32=Yes] What was the maximum diving depth during this vacation?

**Part III: Lifestyle**

1. Did anything change in your sleeping pattern during your vacation, compare to home? Multiple options can be selected
   1. Yes, I slept more
   2. Yes, I slept less
   3. Yes, I went to sleep later
   4. Yes, I went to sleep earlier
   5. Yes, I woke up earlier
   6. Yes, I woke up later
   7. No, my sleep pattern remained unchanged
2. Did anything change in your eating pattern during your vacation?
   1. Yes, I ate more
   2. Yes, I ate less
   3. Yes, I ate more irregular
   4. No, my eating pattern remained unchanged
3. Did anything change in the intake of the following products:
4. Carbohydrates (for example, potatoes, rice, pasta) [More/Less/Equal/I do not recall]
5. Protein (for example, fish, meat, dairy, eggs or beans) [More/Less/Equal/I do not recall]
6. Alcohol [More/Less/Equal/I never take alcoholic beverages/I do not recall]

**Page: Effects of temperature and physical activity**

1. Have you been more or less physically active during your vacation, compared to your physical activity level at home?
   1. More active than at home
   2. Less active than at home
   3. Equally active as at home
   4. I do not recall
2. [If Q19 = Yes, I clearly experienced less Parkinson’s symptoms during this vacation]: You indicated that you clearly experienced less Parkinson’s symptoms during your vacation. Do you think that the temperature was of influence on this decrease of symptoms?

- Yes, I experienced less Parkinson’s symptoms because of the warm temperatures
  1. Yes, I experienced less Parkinson’s symptoms because of the cool temperatures
  2. No
  3. I do not recall

1. [If Q19 = Yes, I clearly experienced more Parkinson’s symptoms during this vacation] You indicated that you clearly experienced more Parkinson’s symptoms during your vacation. Was the temperature of influence on this increase of your symptoms?

- Yes, I experienced more Parkinson’s symptoms because of the warm temperatures
- Yes, I experienced more Parkinson’s symptoms because of the cool temperatures
- No, the temperature had no influence
- I do not recall

**Page: The effects of stress**

1. Did you experience more or less stress during your vacation compared to your stress level at home?
   1. More stress than at home
   2. Less stress than at home
   3. Equal amount of stress level as at home
   4. I do not recall

The following questions are a validated way to evaluate the effect of stress.

1. [PSS] The questions in this scale ask you about your feelings and thoughts during your vacation. In each case, you will be asked to indicate by circling *how often* you felt or thought a certain way.

*Please* ***tick one box*** *for each question*

[Never/almost never/Sometimes/fairly often/ very often]

1. become upset because of something that happened unexpectedly?
2. feel that you were unable to control the important things in life?
3. feel nervous and stressed?
4. feel confident about your ability to handle your personal problems?
5. feel that things were going your way?
6. find that you could not cope with all the things that you had to do?
7. feel you were able to control irritations in your life?
8. feel you were on top of things?
9. feel angered because of things that were outside of your control?
10. feel difficulties were piling up so high that you could not overcome them?

**Medication**

1. Do you use medication for your Parkinson’s disease? Please note, this question is only about medication to treat symptoms of Parkinson's disease. We do not mean medication that you might use for other illnesses.
   1. Yes
   2. No
2. [If PD medication is used]: Did you change your Parkinson’s medication intake within four weeks prior to, or during your vacation?
   1. Yes, I used more Parkinson’s medication
   2. Yes, I used less Parkinson’s medication
   3. No, there was no change in my Parkinson’s medication intake
3. [If PD medication is used]: Did you notice a change in effectiveness of your Parkinson’s medication?
   1. Yes, my medication was more effective during my vacation
   2. Yes, my medication was less effective during my vacation
   3. No, there was no change in effectiveness of my Parkinson’s medication

**About your earlier vacation experiences**

*[ if option Both surveys are selected]***Please note that here the second part of the survey begins.**
The questions of the first part are very similar to the questions that follow now. This part of the survey is regarding your vacation experiences from a **previous vacation in the period of June 2016 - June 2018**. Please think back to a vacation in this period and answer the rest of these questions with this vacation in mind.

You indicated that you went on vacation in the period between June 2016 - June 2018. Please think back on a vacation you had in this period. This can be a longer vacation, or a weekend, in which you did not sleep at home for at least two consecutive nights. The following questions are regarding this vacation.

**Earlier vacation experiences (June 2016 – June 2018)**

1. What was your vacation destination? Please name the city, state (if applicable) and country. If you have visited multiple vacation destinations (for example, if you visited multiple cities in one trip), you can fill out multiple destinations.
   When you stayed at the same vacation destination the entire vacation, just name the place, (province/state if applicable,) and country for “destination 1” only.
2. During what period did you go on vacation? DD-MM-2018 / DD-MM-2018
3. What was the average temperature during this vacation? [number] °C/F.
4. In what kind of environment did you stay during this vacation? Multiple answers are possible (for example, if you have visited multiple places).
   1. Mountains
   2. Beach
   3. City
   4. Forest
   5. Countryside
   6. Other, namely: …………………

**Page: your Parkinson’s symptoms**

1. Did you, or someone else who was with you during this vacation, notice if your Parkinson’s symptoms changed during the vacation, compared to how you normally experience your symptoms at home? Please do not only think of differences in motor symptoms, such as tremor, slowness, balance, difficulty walking or fine motor skills (such as getting dressed or writing), but also report on non-motor symptoms such as pain, mood or sleep and your overall functioning.
   1. Yes, I clearly experienced more Parkinson’s symptoms during my vacation.
   2. Yes, I clearly experienced less Parkinson’s symptoms during my vacation.
   3. No, I did not experience any or only very small differences in my Parkinson’s symptoms during my vacation.
2. We will now ask a couple of questions about changes in specific Parkinson’s symptoms, which you or someone close to you may have noticed changed during your vacation. Below you will find a list of symptoms. Indicate for every symptoms to what extent the symptom was different during the vacation, compared to how you normally experience that symptom at home. If you do not recall a particular symptom or a symptom is not applicable because you normally don’t experience it, please indicate so in your answers.

Did you experience changes in: [Yes, I experienced this symptom less/Yes I experienced this symptom more/No, this symptom did not change/I do not recall/Not applicable because I do not experience this symptom normally]

- 1. Tremor
  2. Gait problems
  3. Balance problems
  4. Difficulties with fine motor skills (i.e., writing, getting dressed)
  5. Pain or muscle cramps
  6. Uncontrolled movements (no tremor)
  7. Stiffness of the muscles
  8. Overall slowness

1. We are also interested in changes of health problems, which you or someone close to you may have noticed during your vacation. Please indicate for each health problem listed below to what extent it was different during the vacation, compared to how you normally experience that problem at home.[yes I experience this health problem less/yes, I experience this health problem more/ No this health problem was not different / I do not recall/ Not applicable because I do not have this health problem normally]
   1. General restrictions of daily activities
   2. Difficulty sleeping
   3. Changes in mood
   4. Fatigue
2. [if at least one item is scored as more/less/better/worse in the previous 2 questions]: You indicated to have experienced a change in at least one symptom or health problem during the vacation. Can you please describe which change(s) you experienced in your own words? Be as detailed as possible.
3. What do you think caused this change in your Parkinson’s symptoms? [Open]
4. To what extent was the environment you stayed in, for example, the beach, mountains or a city, of influence on the changes you experienced?()

- a very great extent
- a great extent
- some extent
- Not at all
- I do not recall

1. You indicated that the environment you were in was of influence on the changes in Parkinson’s symptoms. Can you please describe, in your own words, why you think so.[open]
2. How long did this/these change(s) typically last?
   1. 0-30 minutes
   2. 30-60 minutes
   3. Several hours
   4. A day
   5. Several days or longer
   6. Variable duration
3. Have you experienced these changes just once, or multiple times while you were on vacation?
   1. Once
   2. It happened more often
   3. I do not recall

[If destination = Mountains] You indicated earlier that you visited the mountains during your vacation. The next questions are about the time that you spent in the mountains:

1. [if destination= mountains] To what extent did your Parkinson’s symptoms change when you stayed in the mountains, compared to how you experience your symptoms at home?
   1. I experienced a clear increase of my symptoms
   2. I experienced a clear decrease of my symptoms
   3. I did not experience a clear difference
   4. I do not recall
2. [If previous question = yes] You indicated that you experienced a difference in symptoms while staying in the mountains. Can you please describe, in your own words, what your experiences were? Be as detailed as possible. [open]
3. During your vacation, have you been at an altitude above 6500 feet? It does not matter if this was a short or long stay. Passing through a high mountain pass by car already is sufficient. A longer stay at high altitude is also possible.
   1. Yes, I was at an altitude of 6500 feet or higher
   2. Yes, I was at an altitude of 6500 feet or higher, but only on an airplane
   3. No, I have not been at an altitude above 6500 feet
4. [if previous question = Yes (option 1)] You indicated that you were at 6500 feet or above. Did you experience a change in Parkinson’s symptoms while staying at this high altitude?

- Yes, symptoms increased
- Yes, symptoms decreased
- No, high altitude does not have an effect on my Parkinson’s symptoms
- I do not recall

1. [if destination = beach] Previously you indicated that you went to the beach. Did you go scuba diving during your stay at the beach?

- yes, I went scuba diving
- No, I did not go scuba diving

1. [If previous question=Yes] Did you experience a change in Parkinson’s symptoms when you went scuba diving?

- Yes, my symptoms increased
- Yes, my symptoms decreased
- No, I did not experience a difference

1. [If Q64=Yes] What was the maximum diving depth ?

**Part V: To conclude**

1. These were all the questions regarding vacation experiences and changes in your Parkinson’s symptoms. If you have any important additions or experiences that have not been addressed in this survey, you can report these here.[open].
2. With whom did you complete this survey?
   1. Alone
   2. Together with the person that was with me during the vacation(s)
