# Supplementary material for: How Vacations Affect Parkinson's Disease
Source: Mov Disord Clin Pract. 2022 Nov 2;10(1):151–3. doi: 10.1002/mdc3.13597 (PMC9847288; doi:10.1002/mdc3.13597)
Supplement: Supplementary file 2 — Supplementary Materials 2. Demographics and Vacation Characteristics [file MDC3-10-151-s002.docx]

# Supplementary Materials 2: Demographics and vacation characteristics

Participants resided in 21 different countries, with the highest percentage of respondents from the Netherlands (65.3%), Sweden (10.2%) and the United States (6.1%). Vacations lasted for a median of 10 days, ranging from 2 days to 108 days. The median (self-reported) peak temperature during the vacation was 25 degrees Celsius (range -7 to 38). Participants mainly spent their vacation in the Netherlands (24.5%), Spain (11.6%) or France (8.8%). 15.7% went on vacation at high altitude (>2000 meters, i.e. > 6500 ft).

**Table S1:** Population characteristics, country of origin and destination (n=147)

| Variable | Value |
| --- | --- |
| Age (years, median (range)) | 63 (40-78) |
| Gender (women, n (%)) | 73 (49.7%) |
| Quality of Life (PDQ-8, median (range)) | 8 (0-24) |
| Stress (PSS, median (range))^1^ | 16 (7-24) |
| Disease duration (years, median (range)) | 6 (0-42) |
| PD-medication use (yes, n (%)) | 138 (93.9%) |

^1^ Higher scores indicating more stress

| Recent vacatioN | Country of origin | destination |
| --- | --- | --- |
| Australia | 3 (2.0%) | 2 (1.4%) |
| Austria |  | 3 (2.0%) |
| Belgium |  | 2 (1.4%) |
| Bosnia |  | 1 (0.7%) |
| Canada | 1 (0.7%) | 1 (0.7%) |
| Croatia |  | 1 (0.7%) |
| East see region |  | 1 (0.7%) |
| France | 1 (0.7%) | 13 (8.8%) |
| Germany | 1 (0.7%) | 6 (4.1%) |
| Greece | 1 (0.7%) | 6 (4.1%) |
| Iceland | 1 (0.7%) |  |
| India |  | 1 (0.7%) |
| Indonesia |  | 2 (1.4%) |
| Iran | 7 (4.8%) | 5 (3.4%) |
| Ireland | 3 (2.0%) |  |
| Italy | 1 (0.7%) | 5 (3.4%) |
| Korea |  | 1 (0.7%) |
| Mexico |  | 1 (0.7%) |
| Morocco |  | 1 (0.7%) |
| Netherlands | 96 (65.3%) | 36 (24.5%) |
| Norway |  | 3 (2.0%) |
| Pakistan | 1 (0.7%) |  |
| Portugal |  | 2 (1.4%) |
| Scotland |  | 3 (2.0%) |
| Slovenia |  | 1 (0.7%) |
| South Africa | 1 (0.7%) | 1 (0.7%) |
| Spain | 1 (0.7%) | 17 (11.6%) |
| Sweden | 15 (10.2%) | 7 (4.8%) |
| Switzerland |  | 2 (1.4%) |
| Thailand |  | 1 (0.7%) |
| Turkey |  | 1 (0.7%) |
| United Kingdom | 5 (3.4%) | 7 (4.8%) |
| Unknown |  | 6 (4.1%) |
| United States | 9 (6.1%) | 8 (5.4%) |
