# Supplementary material for: How Vacations Affect Parkinson's Disease
Source: Mov Disord Clin Pract. 2022 Nov 2;10(1):151–3. doi: 10.1002/mdc3.13597 (PMC9847288; doi:10.1002/mdc3.13597)
Supplement: Supplementary file 3 — Supplementary Materials 3. Symptom Changes, Changes in Contextual Factors, and Determinants of Symptomatic Worsening [file MDC3-10-151-s003.docx]

# Supplementary Materials 3: Symptom changes, changes in contextual factors and determinants of symptomatic worsening

**Figure S1**: Self-reported change in global and specific Parkinson’s symptoms during a vacation (n=147).


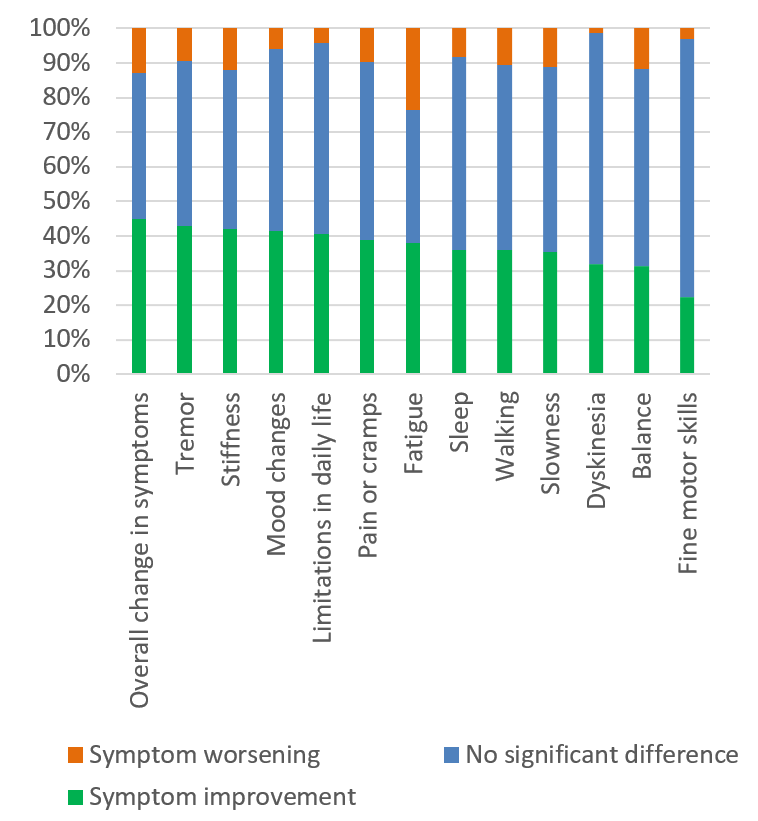


**Table S2:** Self-reported change in contextual factors while on vacation (n=147). Data are presented as the absolute number of individuals who reported a change in the determinant, with the relative group size presented between brackets.

| **Determinant** | **Less** | **Equal / unchanged** | **More** |
| --- | --- | --- | --- |
| Stress | 71 (50.7%) | 51 (36.4%) | 18 (12.9%) |
| Physical activity | 21 (14.6%) | 35 (24.3%) | 88 (61.1%) |
| Sleep | 18 (12.2%) | 80 (54.4%) | 49 (33.3%) |
| High altitude | N/A | 126 (85.7%) | 21 (14.3%) |
| Medication | 8 (5.8%) | 117 (85.4%) | 12 (8.8%) |
| Proteins | 15 (11.6%) | 75 (58.1%) | 39 (30.2%) |
| Carbohydrates | 21 (16.5%) | 81 (63.8%) | 25 (19.7%) |
| Alcohol | 10 (8.9%) | 52 (46.4%) | 50 (44.6%) |

**Table S3:** Effect estimates for potential determinants of symptomatic worsening. For every association, a percentile point difference (Δ%) and odds ratio (OR, with 95% CI) is given. Δ% reflects the percent point difference of symptom worsening between the group that was exposed to the potential determinant and the group with unchanged exposure to the potential determinant (compared to the at-home situation). Statistically significant findings are sorted by their magnitude in shades of red.

|  | **Less physical activity** *(n=21)* | | | | **Less sleep** *(n=18)* | | | | **More stress** *(n=18)* | | | |
| --- | --- | --- | --- | --- | --- | --- | --- | --- | --- | --- | --- | --- |
|  | **Δ %** | OR | CI | | **Δ %** | OR | CI | | **Δ %** | OR | CI | |
| **Primary outcome**  Global symptoms | 20.0 | **5.1** | 1.1 | 24.1 | 16.7 | 3.0 | 0.8 | 11.5 | 25.2 | **3.7** | 1.0 | 13.1 |
| **Secondary outcomes** |  | | | | | | | | | | | |
| Tremor | 10.2 | 2.4 | 0.3 | 17.5 | 3.3 | 1.9 | 0.1 | 25.3 | 32.4 | **21.5** | 2.0 | 231.6 |
| Walking | 30.9 | **14.5** | 1.4 | 151.3 | 17.1 | 3.7 | 0.7 | 18.6 | 33.2 | **7.9** | 1.6 | 39.7 |
| Balance | 13.5 | 2.2 | 0.4 | 11.1 | 14.5 | 3.9 | 0.6 | 24.4 | 38.8 | **12.7** | 2.3 | 69.7 |
| FMS | 17.6 | NA |  |  | -4.2 | NA |  |  | 6.3 | NA |  |  |
| Pain | 14.4 | 3.9 | 0.6 | 24.7 | 10.2 | 2.4 | 0.5 | 12.7 | 34.7 | **6.9** | 1.6 | 30.2 |
| Dyskinesia | -6.3 | NA |  |  | -4.0 | NA |  |  | -4.3 | NA |  |  |
| Stiffness | 27.1 | **9.0** | 1.5 | 54.3 | 3.3 | 1.4 | 0.2 | 9.0 | 30.1 | **5.4** | 1.3 | 21.7 |
| Slowness | 28.6 | **6.4** | 1.1 | 38.8 | 17.1 | 5.3 | 0.7 | 38.9 | 40.4 | **16.2** | 3.0 | 88.4 |
| LDL | 18.8 | NA |  |  | 3.9 | 2.4 | 0.1 | 43.3 | 17.5 | 9.0 | 0.8 | 106.8 |
| Sleep | 9.7 | 2.7 | 0.5 | 15.0 | 23.1 | **>100** | >100 | >100 | 39.3 | **16.9** | 2.8 | 102.0 |
| Mood | -4.0 | 1.1 | 0.1 | 17.2 | 24.2 | **13.6** | 1.1 | 172.6 | 21.4 | 7.0 | 0.9 | 52.9 |
| Fatigue | 12.7 | 1.5 | 0.4 | 5.7 | 49.4 | **15.2** | 3.4 | 67.1 | 60.4 | **17.6** | 4.1 | 75.6 |

FMS = fine motor skills; LDL = limitations in daily life; NA = not available (insufficient number of individuals)**.**
