# Supplementary material for: How Vacations Affect Parkinson's Disease
Source: Mov Disord Clin Pract. 2022 Nov 2;10(1):151–3. doi: 10.1002/mdc3.13597 (PMC9847288; doi:10.1002/mdc3.13597)
Supplement: Supplementary file 4 — Supplementary Materials 4. Principal Component Analysis and Visualization of Study Results [file MDC3-10-151-s004.docx]

# Supplementary Materials 4: Principal component analysis and visualization of study results

**Table S4:** Principal component analysis (for individuals with net improvement of symptoms on vacation)

| *Principal components* | | | | |
| --- | --- | --- | --- | --- |
|  |  | | | |
|  | Medication (dys)balance | Dopaminergic  state | Physical and mental load | (Over)activity |
| Mood changes | ,**860** |  | ,317 |  |
| Dyskinesia | ,**833** |  |  |  |
| Limitations in daily life | ,**689** | ,505 |  |  |
| Balance problems | ,**688** | ,556 | ,336 |  |
| Tremor | ,622 |  | ,361 |  |
| Slowness |  | **,787** |  |  |
| Fine motor skills |  | **,749** |  |  |
| Walking problems | ,513 | ,**671** | ,376 |  |
| Sleep problems |  |  | **,821** |  |
| Fatigue |  |  | **,805** | ,513 |
| Pain or cramps |  |  |  | **,854** |
| Stiffness |  |  |  | **,827** |


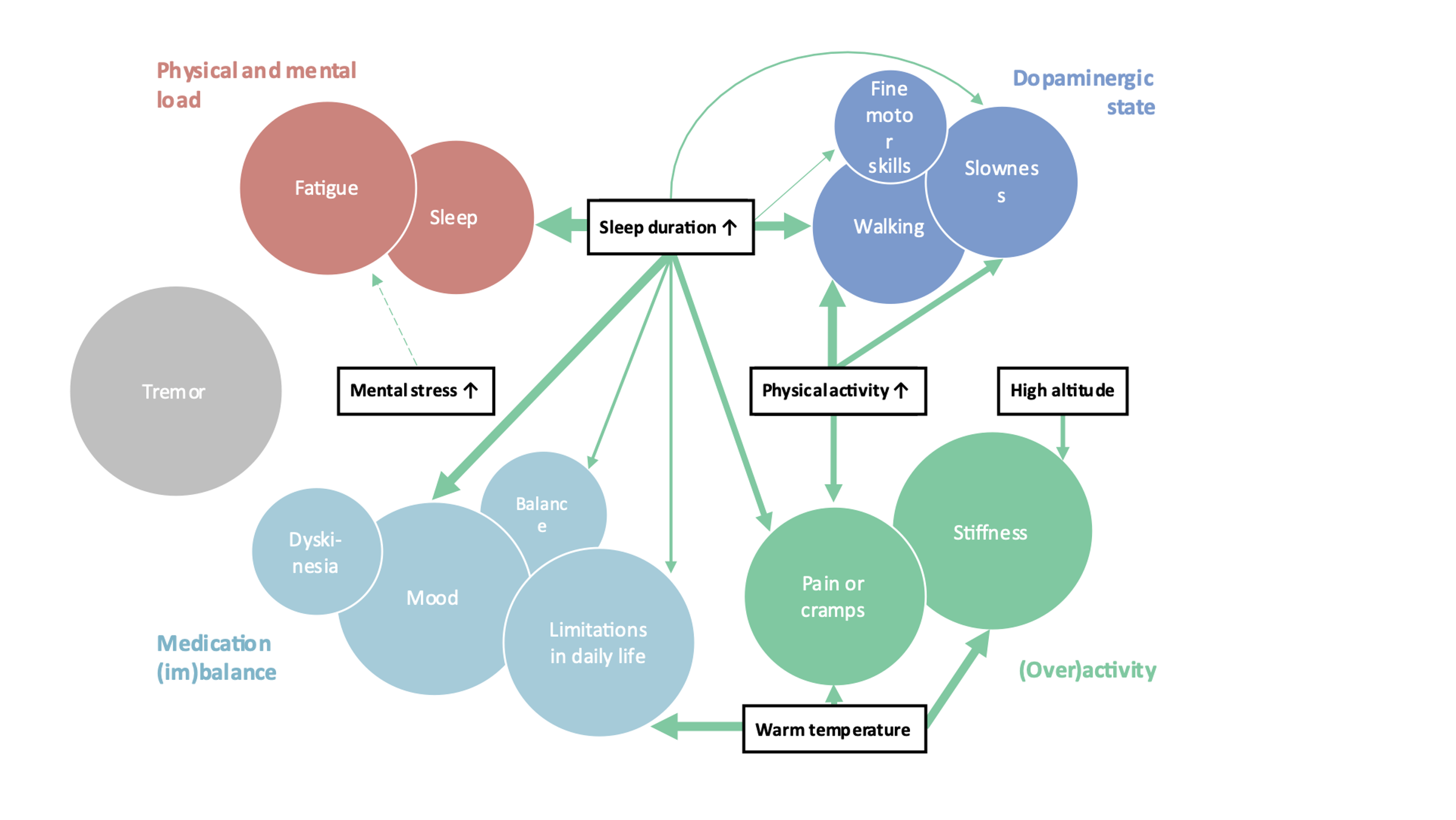
**Figure S2:** Association between contextual factors (boxes) and PD symptom improvement (circles). Circle magnitude reflects group-level percentage of symptomatic improvement (Figure 1). Arrow thickness reflects the OR of significant associations between symptomatic improvement and any contextual factor (Table 3); dashed line indicates a trend. Symptoms are clustered by PCA (Supplementary) using groups of colored circles.
